# Effectiveness of telerehabilitation in the management of adults with stroke: A systematic review

**DOI:** 10.1371/journal.pone.0225150

**Published:** 2019-11-12

**Authors:** Emma Appleby, Sophie Taylor Gill, Lucinda Kate Hayes, Tessa Lauren Walker, Matt Walsh, Saravana Kumar

**Affiliations:** University of South Australia, Adelaide, South Australia, Australia; University of Mississippi Medical Center, UNITED STATES

## Abstract

**Background:**

Stroke is a leading cause of mortality and morbidity and access to timely rehabilitation can reduce morbidity and help patients to return to normal life. Telerehabilitation can deliver rehabilitation services with the use of technology to increase patient options, deliver services more efficiently and overcome geographical barriers to healthcare access. Despite its popularity, there is conflicting evidence for its effectiveness. Therefore, the aim of this systematic review was to update the current evidence base on the effectiveness of telerehabilitation for stroke.

**Methods:**

A systematic search of databases (Medline Ovid; Embase; Emcare; Scopus; The Cochrane Library; PEDro; OTSeeker) was conducted in April 2018 (updated in October 2018). This review was conducted and reported in line with the Preferred Reporting Items for Systematic Reviews and Meta-Analyses (PRISMA) statement and methodology registered with PROSPERO (CRD42018090445). A modified McMaster critical appraisal tool for quantitative studies was used to assess the methodological quality of the included studies. Given the heterogeneity of the included studies, a descriptive synthesis was conducted.

**Results:**

Out of 1868 studies, 13 randomised controlled trials met the inclusion criteria. A diverse range of interventions were delivered through a variety of telerehabilitation systems and the effectiveness measured through a myriad of outcome measures. Summarised findings from the heterogeneous evidence base indicate that telerehabilitation may have a positive impact on a range of primary and secondary outcomes. However, despite these positive findings, the current evidence lacks clarity and uniformity in terms of intervention parameters and measurement of outcomes, which limits broader application of these results.

**Discussion:**

Telerehabilitation, as an alternate form of rehabilitation for people with stroke, shows potential. However, due to methodological and practical concerns, an unequivocal recommendation cannot be made. Findings from this review may inform future policies and practices regarding the use of telerehabilitation for stroke patients.

## Introduction

Stroke is one of the most common causes of disability and mortality worldwide [1]. In Australia yearly, stroke affects nearly 475,000 people with this number projected to increase to one million by 2050, it also attracts five billion dollars in direct and indirect costs [2]. Due to advances in healthcare and technology, the survival rates for stroke have improved considerably over the past few decades. The overall death rates for stroke in Australia have dropped by 3.7% per year, with the death rate decreasing from 104 deaths per 100,000 people in 1980 to 27 per 100,000 in 2016 [3]. Currently, stroke survivors can access health care through a multidisciplinary stroke unit, which can result in positive outcomes [4]. However, despite this positive evidence, and while 75% of patients with stroke having ongoing rehabilitation needs, only 46% of survivors were referred for rehabilitation in 2017 [5]. This shows that many stroke survivors are missing out on rehabilitation that could improve their function [6].

There are numerous barriers for stroke survivors to access usual face-to-face care such as time restraints, resource limitations, geographical isolation, compliance with rehabilitation, and lack of awareness. One manner of addressing access barriers may be through technological innovation such as telemedicine, and more specifically, telerehabilitation [7]. Telemedicine can be defined as the ‘use of advanced telecommunication technologies to exchange health information and provide health care services across geographic, time, social and cultural barriers’ [7]. This systematic review, as an extension of this definition, defined telerehabilitation as the use of telecommunication, by either direct video or audio, to deliver rehabilitative interventions. This excludes monitoring of patients or general discussions between patients and health professionals, without providing an intervention, and case-conferences as well as merely accessing exercise programs. Patients can access this form of rehabilitation via computer or other devices such as mobile phones or tablets as well [8]. Benefits of telerehabilitation for stroke patients include providing services to remote areas for those isolated from access to services [9] and incorporating more real-life support post-rehabilitation [10].

Previous systematic reviews (SRs) have evaluated the effectiveness of telerehabilitation post-stroke, which have resulted in conflicting findings. Laver et al. [10] identified insufficient evidence to determine the effectiveness of telerehabilitation regarding mobility, participant satisfaction or health-related quality of life in stroke. Chen et al. [4] identified limited moderate evidence supporting telerehabilitation being equally beneficial to usual care in improving motor function and abilities for activities of daily living (ADLs). Therefore, the purpose of this review was to update the literature on the effectiveness of telerehabilitation for stroke which may be used to inform clinical practice and patient decision-making.

## Methods

### Search protocol and registration

The Preferred Reporting Items for Systematic Reviews and Meta-Analyses (PRISMA) framework [11] forms the foundation for the methodology of this systematic review. The PRISMA-P guidelines [12] were used in creating this protocol and the methodology for this review was registered with PROSPERO (CRD42018090445)

### Search strategy

The PICO format was utilised in the development search strategy with search terms and limits relating to population of interest and intervention. The development of the search strategy was informed by discussions with the University of South Australia Academic Librarian. Two reviewers searched a database independently and then cross-referenced results to establish consistency in the search process. If the search results were different, the reviewers met to identify what, if any differences may have arisen and resolved any conflicts through discussion. Once consistency in the search process was achieved, the reviewers commenced formal searching of the databases. In April 2018 the following databases were searched by two independent reviewers (EA, TW): Medline Ovid; Embase; Emcare; Scopus; The Cochrane Library; PEDro; OTSeeker. The searches were conducted in English, from inception to April 2018. This was updated in October 2018. The databases were selected as they have a biomedical and multi-disciplinary focus and contain predominantly peer reviewed literature. The reference lists of relevant literature were also searched to identify potential additional articles (pearling). Grey literature searching through an internet web engine (Google and Google Scholar) was undertaken to identify any additional publications, with the first ten pages of results reviewed. Websites of relevant organisations (such as the National Stroke Foundation) were also searched for further publications.

### Study design

While all forms of primary and secondary research (for pearling purposes) were searched, only randomised control trials (RCTs) were included in this review.

### Population

Studies were included if participants were adults (18 years and over) who had suffered a stroke, with no exclusion based on type. Children (17 years and under) were excluded from this review as the incidence of stroke in this population is low, and the paediatric cohort presents additional challenges and barriers to delivering therapy via technological means. Studies including patients with conditions other than stroke or mixed population groups were excluded unless results were reported separately for each included diagnosis. In this case only results specific to stroke were included. Studies were not excluded based on participants time post stroke or gender, there was also no upper limit placed on the age of participants from included studies.

### Intervention

A broad range of telerehabilitation interventions was included to gain a comprehensive overview of current approaches. For inclusion, the intervention needed to include the provision of rehabilitative services aimed at improving physical function, rather than merely an interaction with healthcare professionals (e.g. case conference, education). The intervention also had to be directed to the stroke patient themselves rather than their caregiver. Telerehabilitation was not limited to a specific mode of technology and studies were included even if telerehabilitation was an adjunct to usual care. Virtual reality or other technology based interventions were not excluded from this review if they were delivered via telecommunication methods and satisfied our other inclusion criteria.

### Outcome measures

Primary outcomes of interest were motor function and activities of daily living. Other outcomes of interest included patient satisfaction and independence. Studies were not included or excluded based on the outcome measures they used with the aim of gaining a broad understanding of the effect telerehabilitation may have.

### Study selection

The results from the searches were exported into the industry standard bibliographic tool Endnote^TM^ to merge search results from different databases. The studies were then transferred to the data management software for systematic reviews, Covidence^TM^ (Covidence.org), where duplicates were removed. Once the initial results list was finalised, five reviewers (EA, SG, LH, TW, MW) each independently screened a selection of the titles and abstracts of studies. Studies were identified to be included based on the established inclusion and exclusion criteria. Following title and abstract screening, the full-texts of the included studies were reviewed based on the inclusion and exclusion criteria. During the selection process, if there were any disagreements, an external reviewer with experience in systematic review process (SK) provided an independent and final decision.

### Methodological quality

To evaluate the methodological quality of the included studies, a modified McMaster Critical Appraisal Tool [13] was used. This tool assessed eight main components of the studies, including: study purpose; background literature review; study design; sample size justification; reliability and validity of outcome measures; description, contamination and co-intervention regarding intervention; statistical significance and analysis methods of results, dropout reporting; and conclusion with implications for practice. The tool uses a rating scale where yes = 1 point and no = 0 points. An overall score for each study was then generated for comparison. Five reviewers (EA, SG, LH, TW, MW) independently assessed the included studies methodological quality and any discrepancies were resolved through discussion. The level of evidence for included studies was determined using the Intervention category of the Australian National Health and Medical Research Council’s (NHMRC) evidence hierarchy [14].

### Data extraction

Customised data extraction forms were developed specifically for this systematic review. Data extracted included study and participants' characteristics, interventions, comparators, outcomes and the overall results. The forms were initially screened by an external reviewer (SK) to ensure all relevant data would be captured and any feedback was incorporated into the final version. Throughout this process the summary measures used throughout this review mirror those used by the individual studies. The data from the included studies was extracted by five reviewers (EA, SG, LH, TW, MW) and any disputes were resolved through discussion. An independent reviewer (SK) was used in cases where the group could not reach a consensus or to clarify any outstanding issues.

### Data synthesis

Due to the generalised heterogeneity of the included studies, a meta-analysis of the studies could not be undertaken. Despite some studies using the same outcome measures (e.g. the Barthel Index scale, Berg Balance Scale, and the Fugl-Meyer Upper Extremity test) a meta-analysis for these specific outcome measures was not feasible as the parameters of the interventions varied greatly. (see S1 Table). Furthermore, participant characteristics differed as well with variability in the type of stroke and post-stroke intervention period.

Due to these limitations, a descriptive synthesis of the results using the NHMRC FORM framework was used to grade and provide a framework to synthesis data from the studies [15]. There are five key components to this framework: 1) Quantity and quality of evidence, 2) Consistency of the study results, 3) Clinical impact, 4) Generalisability, 5) Applicability to the Australian health care setting. The final component was not used in this systematic review, given that the findings may be relevant to an international setting.

## Results

### Search results

**Fig 1:**
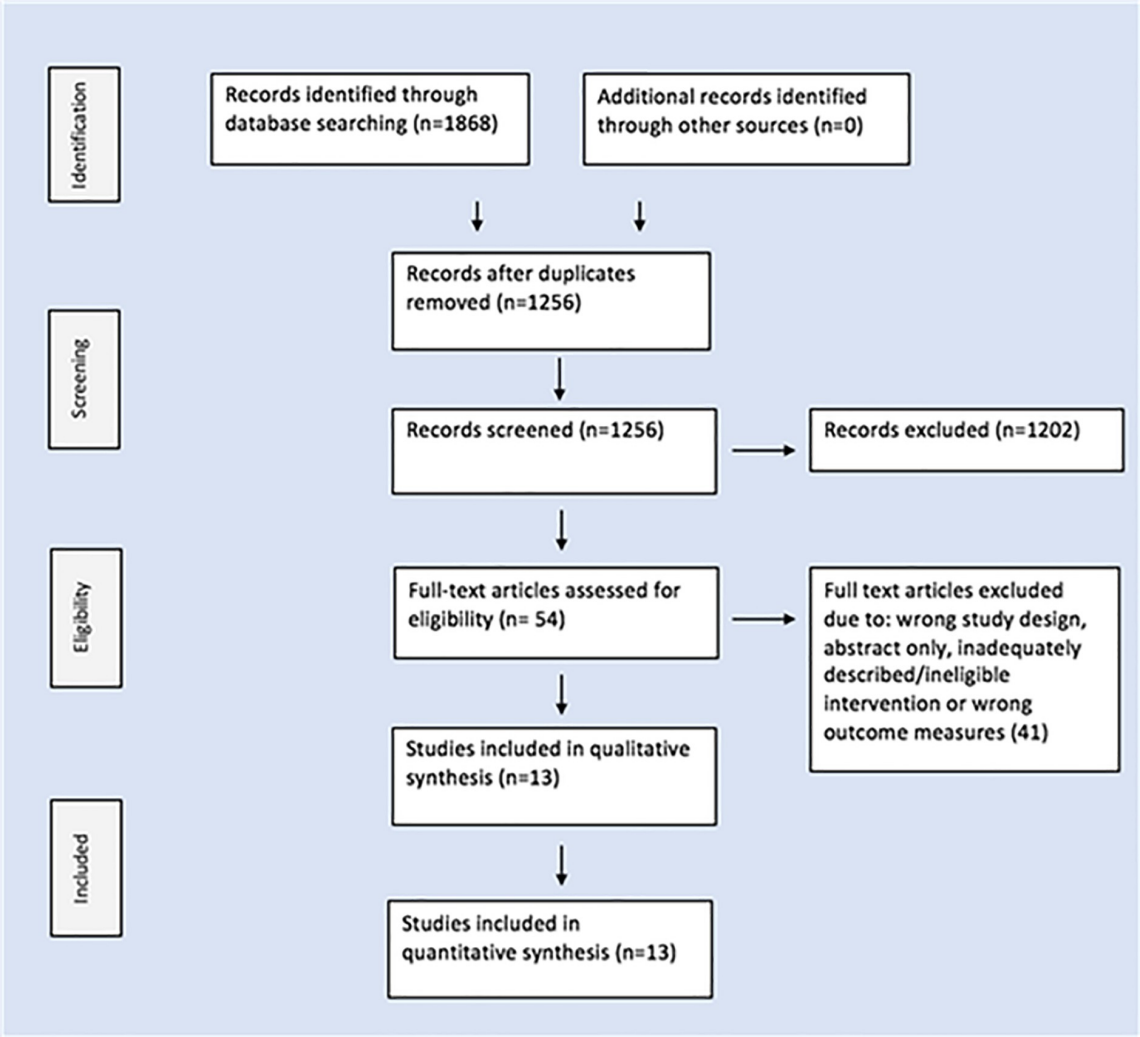
PRISMA flowchart.

The search strategy identified 1868 studies. After the removal of duplicates, 1256 studies were screened for title and abstract. 54 studies progressed to the next stage and were reviewed in full text. 41 were then excluded because of study design (n = 19), abstract only (n = 12), inadequately describe/ineligible intervention (n = 6) or wrong outcome measures (n = 4). 13 studies were consequently included in this review. Two of the included publications, Chumbler et al. 2012 [16] & Chumbler et al. 2015 [17], shared data from the same original study, reporting on different outcomes. The literature selection process is outlined in Fig 1.

### Methodological quality

Table 1 provides a summary of the levels of evidence and critical appraisal scores of all included studies. As per the NHMRC's levels of evidence [14], all 13 studies were classified as RCTs and ranked level II accordingly. The methodological quality of the included studies could be considered as moderate to good with the scores ranging from 7–12 out of a possible 14. The mean score for the studies was 10.38 (standard deviation (SD) 2.01). Two main areas of concern were the potential for co-intervention in all 13 studies and a lack of justification for sample size in 10 studies [18–27]. Other areas of concern included modified psychometric properties of outcome measures lacking validity [24, 26], insufficient background literature, inadequate description of interventions, potential for contamination of results, and poorly supported conclusions.

**Table 1:** Levels of evidence and critical appraisal scores.

Study	1	2	3	4a	4b	4c	5a	5b	6a	6b	6c	7a	7b	7c	7d	8	Total /14	%
**Carey et al. 2007 [18]**	Y	N	RCT–II	20	Y	N	Y	Y	Y	Y	N	Y	Y	Y	Y	Y	11	79
**Chen et al. 2017 [28]**	Y	Y	RCT—II	54	Y	Y	Y	Y	Y	Y	N	Y	Y	Y	Y	Y	13	93
**Chumbler et al. 2012 [16]**	Y	Y	RCT—II	52	Y	Y	Y	Y	Y	Y	N	Y	Y	Y	Y	N	12	86
**Chumbler et al. 2015 [17]**	Y	Y	RCT—II	52	Y	Y	Y	Y	Y	Y	N	Y	Y	Y	Y	Y	13	93
**Deng et al. 2012 [19]**	Y	Y	RCT—II	19	Y	N	Y	Y	Y	Y	NAD	Y	Y	Y	Y	Y	12	86
**Forducey et al. 2012 [20]**	Y	Y	RCT—II	11	N	N	Y	Y	N	NAD	NAD	Y	N	Y	Y	N	6/13	50
**Huijgen et al. 2008 [21]**	Y	Y	RCT—II	16	Y	N	Y	Y	N	NAD	NAD	N	N	Y	Y	N	7	50
**Kizony et al. 2013 [22]**	Y	Y	RCT—II	20	Y	N	Y	Y	Y	Y	N	Y	Y	Y	Y	Y	12	86
**Krpic, Savanovic & Cikajlo 2013 [23]**	Y	Y	Pilot RCT—II	26	Y	N	Y	Y	Y	N	NAD	Y	Y	Y	N	N	9	64
**Lin et al. 2014 [24]**	Y	Y	Pilot RCT—II	24	Y	NA	NAD	Y	Y	NAD	NAD	Y	Y	Y	Y	N	9	64
**Llorens et al. 2015 [25]**	Y	N	RCT—II	31	Y	N	Y	Y	Y	NAD	N	Y	Y	Y	Y	Y	10	71
**Piron et al. 2008 [26]**	Y	Y	Pilot RCT—II	10	N	N	Y	Y	N	Y	NAD	Y	Y	N	Y	Y	11	79
**Piron et al. 2009 [27]**	Y	Y	RCT—II	36	Y	N	Y	Y	N	Y	NAD	Y	Y	Y	N	N	9	64

McMaster items to be scored: 1. Was the purpose stated clearly?; 2. Was relevant background literature reviewed?; 3a. What was the study design?; 4a. Sample number; 4b. Was the sample described in detail?; 4c. Was the sample size justified?; 5a. Were the outcome measures reliable?; 5b. Were the outcome measures valid?; 6a. Intervention was described in detail?; 6b. Contamination was avoided?; 6c. Cointervention was avoided?; 7a Results were reported in terms of statistical significance?; 7b. Were the analysis method/s appropriate?; 7c. Clinical importance was reported?; 7d. Drop-outs were reported?; and 8. Conclusions were appropriate given study methods and results?. Y = yes, N = No, NAD = not addressed.

### Study characteristics

The study characteristics are outlined in Table 2. The 13 RCTs published between 2007 and 2017. The studies were conducted in a number of countries including Belgium [21], Italy [26, 27], Spain [25], Taiwan [24], USA [16–20], China [28], Slovenia [22, 23].

**Table 2:** Study characteristics.

Author Sample Size (Drop outs)	Age	Type of stroke	Intervention	Comparator	Outcome Measures
Carey et al. 2007 [18] 20 (0)	**Track group:** 65.9 ± SD 7.4 **Move group:** 67.4 ± SD 11.8	8 cortical, 12 subcortical strokes	Track group: Customised software for tracking finger and wrist movement used in own home. Delivered via TR.	Move group: As with intervention, but focus on movement with no specific tracking (therefore lack of knowledge of results or performance). Delivered via TR.	B&B, JTT, Finger ROM, fMRI
Chen et al. 2017 [28] 54 (4)	**TRG:** 66.5 SD ± 12.1 **CG:** 66.2 SD ± 12.3	**TRG:** Type of stroke: Ischaemic: 24 Hemorrhagic: 3 **CG:** Type of stroke: Ischaemic: 22 Hemorrhagic: 5	Exercise sessions via video and neuromuscular stimulation	As with intervention, but in outpatient department.	MBI, BBS, MRS, CSI and RMS
Chumbler et al. 2012 and 2015 [16, 17] 52 (9)	**TRG:** 67.1 SD ± 9.5 **CG:** 67.7 SD ± 10	Type not specified No cognitive impairments	Functional mobility therapy, in-home messaging device monitored and usual care as required.	Usual care was provided via veterans association or non-veteran association care accessed at the discretion of the patient.	FONEFIM & LLFDI
Deng et al. 2012 [19] 19 (3)	Median (Q1, Q3) **Track group:** 52 (47, 60) **Move group:** 58 (54, 64)	1 cortical, 8 subcortical, 7 cortical/subcortical strokes Cognition appropriate to follow commands	Customised software for ankle movement. Delivered by TR.	Attempted same frequency as intervention, movement not tracked. Delivered by TR.	GA, 10MWT, fMRI
Forducey et al. 2012 [20] 16 (5)	Mean age of total participants: 60	Type not specified	Education sessions aimed at self-care, mobility and posture delivered via desktop videophone using standard telephone lines.	As with intervention but provided face to face.	FIM, SF-12
Huijgen et al. 2008 [21] 16 (4)	**TRG:** 69 SD ± 8 **CG:** 71 SD ± 7	Stroke type not reported	Training via the telerehabilitation system: Home Care Activity Desk (HCAD) for 4 weeks	Usual care and completion of generic exercises prescribed by doctor	ARAT, NHPT, VAS for user satisfaction
Kizony et al. 2013 [22] 20 (2)	**TRG:** 59.3 SD ± 11.3 **CG:** 54.6 SD ± 13.6	Type not specified	3D video camera software in simulated home environment. Patient controls movements in games.	Independent arm exercises	FMUE, CAHAI & MAL
Krpic, Savanovic and Cikajlo 2013 [23] 26 (NR)	**CG:** 63 SD ± 8.5 **Non TR Balance Trainer group:** 61 SD ± 7.4 **TR:** 58.5 SD ± 12.1	Type not specified Cognition appropriate to follow commands	Training using the independently designed ‘Balance Trainer device.’	Conventional balance training: face to face training with a physiotherapist Non TR Balance Trainer intervention: Using Balance Trainer device in outpatient setting.	BBS, TUG, 10MWT, Change in specific virtual reality parameters including task time and collisions, resource analysis
Lin et al. 2014 [24] 24 (0)	**TRG:** 74.6 SD ± 2.3 **CG:** 75.6 SD ± 3.4	Type not specified Cognition appropriate to follow commands	Face to face in person standing balance training plus TR via wireless sensor network to train balance for an unspecified frequency	Face to Face standing balance training No TR	BBS, BI & TRSQ
Llorens et al. 2015 [25] 31 (1)	**TRG:** 55.5 SD ± 9.6 **CG:** 55.6 SD ± 7.3	19 hemorrhagic, 11 ischemic strokes	TV, computer and Microsoft Kinect used to train balance as well as usual care for motor function. Also received conventional therapy	Physiotherapist monitored Microsoft Kinect in clinic while assisting with other patients. CG also received conventional therapy.	BBS, POMA-B, POMA-G, BBA, SUS, IMI, Cost in dollars.
Piron et al. 2008 [26] 10 (0)	**TVR:** 53 SD ± 15 **HVR:** 65 SD ± 11	Ischemic stroke in MCA No cognitive impairments	TVR group = 3D motion tracking system, therapist created tasks in VR and videoconferencing.	HVR group = The same 3D motion tracking system in hospital with therapist present.	FMUE scale MDSQ
Piron et al. 2009 [27] 36 (NR)	**TRG:** 66 SD ± 7.9 **CG:** 64.4 SD ± 7.9	Ischemic stroke in MCA No cognitive impairments	3D motion tracking system generated VR environment with motor tasks.	Usual UL therapy	FMUU, ABILHAND, AS

**Key:** CG = Control Group, HVR = Hospital Virtual Reality, MCA = middle cerebral artery, SD = Standard Deviation, TR = Telerehabilitation, TRG = Telerehabilitation Group, TVR = Tele Virtual Reality.

**Outcome measures**:

**Motor Function:** Fugl-Meyer scale (FM), Fugl-Meyer Upper Extremity score (FMUE), ABILHAND scale (ABILHAND), Box and Block test (B&B), Jebsen Taylor test (JTT), finger movement tracking test (Finger tracking), Berg Balance Scale (BBS), Gait assessment (GA), Ten-meter walk test (10MWT), Motor subscale of the Functional Independence Measure (mFONEFIM), Berg Balance Scale (BBS), The performance-oriented mobility assessment balance subscale (POMA-B), The performance-oriented mobility assessment gait subscale (POMA-G), Brunel Balance Assessment (BBA), Timed Up and Go (TUG), Action Research Arm Test (ARAT), Nine Hole Peg Test (NHPT)

**ADLs**: Motor Activity Log (MAL), Barthel Index for functional activity (BI), Modified Barthel Index (MBI), Chedoke Arm and Hand Activity Inventory (CAHAI)

**Satisfaction/Quality of Life**: Multidimensional disease and treatment specific satisfaction questionnaire (MDSQ), Self-developed Tele-rehabilitation satisfaction questionnaire (TRSQ), Hospital Dimension of the Stroke-Specific Patient Satisfaction with Care (SSPSC-Hospital), Home Dimension of the Stroke-Specific Patient Satisfaction with Care (SSPSC-Home), The system usability scale (SUS), Care-giver strain index (CSI), Visual Analogue Scale (VAS), Short Form 12 (SF-12)

**Independence/Self-efficacy:** The Falls Efficacy Scale (FES), Intrinsic motivation inventory (IMI), Late-Life Function and Disability Instrument (LLFDI), Modified Rankin Scale 7 for disability (MRS).

**Miscellaneous:** finger range of motion tracking (finger ROM tracking), root square mean of target muscles (RMS), Ashworth Scale (AS)

### Participant characteristics

The number of participants in any single study varied from 10–81 with an age range from 28–85 years and a gender bias towards men (more male participants than women). Commonly reported characteristics of the participants were the type of stroke, affected side and time since stroke. In nine of the 13 studies, participants were excluded if they had cognitive impairments which limited their comprehension [16–19, 21, 24–27].

### Types of intervention

While telerehabilitation was a common mode of delivery of interventions, the actual interventions that were delivered varied within the included studies. Intervention frequency and duration also varied ranging from daily sessions to three times per week, and lasted from ten days to 12 weeks. Four of the 13 studies had each session supervised via a telerehabilitation system [22, 24, 26, 27]. The remaining nine studies included partial supervision through telerehabilitation systems and physical assistance [16–21, 23, 25, 28]. There was a range of telerehabilitation systems used but the common elements included video and audio equipment for videoconferencing capabilities. Eight studies used videoconferencing for instruction and communication [16–21, 24, 25]. Additionally, three studies used 3D motion equipment and software to generate virtual representations of participants’ movements [22, 26, 27]. Chen et al.[28] combined videoconferencing with biofeedback and physiological data from participants. S1 Table provides an overview of the parameters of intervention.

### Outcomes

Table 3 summarises the outcomes of motor function, ADLs, independence, satisfaction/ quality of life and other miscellaneous outcomes for the included studies.

**Table 3:** Summary of outcomes.

Study	Primary Outcomes				Other outcomes					
	Motor Function		ADLs		Independence/ Self-efficacy		Satisfaction/ Quality of Life		Miscellaneous	
	W	B	W	B	W	B	W	B	W	B
Carey et al. 2007 [18]	↓* + (JTT) B&B ↑* + Finger tracking ↑ * +	JTT ↓+ B&B ↓* - Finger tracking ↑ +							Finger ROM tracking ↑ * +	Finger ROM tracking ↑ * +
Chen et al. 2017 [28]	BBS ↑ +	BBS ↓-	MBI ↑ * +	MBI ↑ +	MRS ↑ +	MRS ↓-	CSI ↓ * +	CSI ↓+	RMS (ECRL) ↑ * + RMS (TA) ↑ * +	RMS (ECRL) ↓- RMS (TA) ↓-
Chumbler et al. 2012 [16]	mFONEFIM ↑ +	mFONEFIM ↑ +			LLFDI ↑ +	LLFDI ↑ +				
Chumbler et al. 2015 [17]					FES ↑ +	FES ↑ +	SSPSC-Hospital ↑ * + SSPSC-Home ↑ +	SSPSC-Hospital ↑ * + SSPSC-Home ↑ +		
Deng et al. 2012 [19]	GA ↑ * + 10MWT ↓ +	GA ↑ * + 10MWT ↑ -								
Forducey et al. 2012 [20]					FIM ↑ * +	FIM ↑ +	SF-12 ↑ * +	SF-12 ↑ +		
Huijgen et al. 2008 [21]	ARAT ↑ + NHPT NR	ARAT ↓- NHPT NR					VAS NR	VAS NR		
Kizony et al. 2013 [22]	FM ↑ * +	FM ND	CAHAI ND MAL ↑ * +	CAHAI ND MAL ↑ +						
Krpic, Savanovic & Cikajlo 2013 [23]	BBS ↑*+ TUG ↓*+ 10MWT ↓*+	BBS ↓ - TUG ↓ + 10MWT ↓ +								
Lin et al. 2014 [24]	BBS ↑ * +	BBS ↓ -	BI ↑ * +	BI ↑ +			TRSQ NA	TRSQ ND		
Llorens et al. 2015 [25]	BBS ↑ * + POMA-B ↑ * + POMA-G ↑ * + BBA ↑ * +	BBS ↑ + POMA-B ↑ + POMA-G ↑ + BBA ↑ +			IMI NA	IMI ↑ +	SUS NA	SUS ↑ +		
Piron et al. 2008 [26]	FMUE ↑ * +	FMUE ↑ +					MDSQ NA	MDSQ ↑ +		
Piron et al. 2009 [27]	FMUE ↑ * + ABILHAND ↑ +	FMUE ↑ * + ABILHAND ↑ +							AS ↓ * +	AS ↓ +

**Key:** W = within intervention group, B = between intervention and control groups, ND = no difference, NA = not applicable, NR = either not reported by the researchers or the authors did not provide adequate information to drawer conclusions i.e. no baseline measures, ↑ = increase with intervention, ↓ = decrease with intervention, + = positive change, * = results are statistically significant (p<0.05), ? = significance not reported

**Outcome measures**:

**Motor Function:** Fugl-Meyer scale (FM), Fugl-Meyer Upper Extremity score (FMUE), ABILHAND scale (ABILHAND), Box and Block test (B&B), Jebsen Taylor test (JTT), finger movement tracking test (Finger tracking), Berg Balance Scale (BBS), Gait assessment (GA), Ten-meter walk test (10MWT), Motor subscale of the Functional Independence Measure (mFONEFIM), Berg Balance Scale (BBS), The performance-oriented mobility assessment balance subscale (POMA-B), The performance-oriented mobility assessment gait subscale (POMA-G), Brunel Balance Assessment (BBA), Timed Up and Go (TUG), Action Research Arm Test (ARAT), Nine Hole Peg Test (NHPT)

**ADLs**: Motor Activity Log (MAL), Barthel Index for functional activity (BI), Modified Barthel Index (MBI), Chedoke Arm and Hand Activity Inventory (CAHAI)

**Satisfaction/Quality of Life**: Multidimensional disease and treatment specific satisfaction questionnaire (MDSQ), Self-developed Tele-rehabilitation satisfaction questionnaire (TRSQ), Hospital Dimension of the Stroke-Specific Patient Satisfaction with Care (SSPSC-Hospital), Home Dimension of the Stroke-Specific Patient Satisfaction with Care (SSPSC-Home), The system usability scale (SUS), Care-giver strain index (CSI), Visual Analogue Scale (VAS), Short Form 12 Survey

**Independence/Self-efficacy:** The Falls Efficacy Scale (FES), Intrinsic motivation inventory (IMI), Late-Life Function and Disability Instrument (LLFDI), Modified Rankin Scale 7 for disability (MRS).

**Miscellaneous:** finger range of motion tracking (finger ROM tracking), root square mean of target muscles (RMS), Ashworth Scale (AS).

### Primary outcomes–motor function

Motor function was the most commonly measured outcome as it was investigated by 11 studies [16, 18, 19, 21–28]. Significant within group improvements were found in eight studies [18, 19, 22–27]. Of these, three studies reported significant between groups differences, with two favouring the intervention groups [18, 19, 27]. Positive but non-significant effect of intervention was found in four studies [16, 19, 27, 28]. Between group differences were reported in 10 studies [16, 18, 19, 21, 23–28] however these were not significant. One study [22] reported no differences between groups for motor function. There were five instances where between group differences favoured the control groups, albeit these differences were not supported by statistical significance. Exploring these results further indicate that three of these results related to balance as measured using Berg Balance Scale. These findings may suggest for balance traditional models of care may achieve greater positive outcomes.

### Primary outcomes–activities of daily living

Three studies investigated ADLs as an outcome from telerehabilitation [22, 24, 28]. All three studies found statistically significant within group improvements. While these positive findings extended for between groups differences too, these results were not significant. One of these studies [22] also used an additional outcome measure to investigate ADLs (Chedoke Arm and Hand Activity Inventory). Extrapolating results specific to Chedoke Arm and Hand Activity Inventory indicates not statistically significant differences within or between the groups.

### Other outcomes–independence and self efficacy

Independence and self-efficacy were explored in five studies [16, 17, 20, 25, 28]. Only one of these studies [20] reported statistically significant results, however, within group improvements were reported in four studies [16, 17, 20, 28]. Positive between group differences were found by Forducey et al. [20], Chumbler et al. [16], Chumbler et al. [17] and Llorens et al. [25] in favour of telerehabilitation. There was one instance of between group difference in favour of the control [28]. This could be due to the control group receiving proprioceptive neuromuscular facilitation from a qualified, trained therapist, in contrast to the intervention group in which untrained caregivers provided this therapy.

### Other outcomes–patient satisfaction/quality of life

Seven studies examined levels of patient satisfaction [17, 20, 21, 24–26, 28]. Five studies found that patients were generally satisfied with telerehabilitation [17, 24–26, 28]. Significant within group differences were found by Forducey et al. [20], Chumbler et al. [17] and Chen et al. [28], with Chumbler et al. [17] also finding significant between group differences.

### Other outcomes–Miscellaneous

Carey et al. [18], Piron et al. [27] and Chen et al. [28] investigated other miscellaneous outcomes including ROM, power and spasticity. All three studies found significant within group improvements with telerehabilitation. Two studies found positive between group differences favouring the telerehabilitation [18, 27], with one study demonstrating statistical significance [18]. Chen and colleagues [28] only reported statistically significant improvements within group but the between group differences favoured the control group, albeit not supported by statistical significance.

### Summary of results

Table 3 provides a summary of outcomes from the 13 included studies, containing 36 outcome measures broadly divided into five domains. Collectively, despite heterogeneity in the intervention parameters underpinning telerehabilitation and the varied measures of outcomes, it appears that there is consistent evidence to indicate that telerehabilitation may be as effective as usual care for motor function, ADLs, independence and self-efficacy and some miscellaneous outcomes. From a patient point of view, it appears that they too are likely to be satisfied with this mode of rehabilitation.

Across the studies, motor function was the most commonly reported outcome, with consistent evidence to indicate positive within group improvements. Between group changes were also generally positive, although there were not consistently supported with statistically significant findings. The three studies that did show statistically significant between group changes all used a tracking system to provide real time knowledge of results. Interestingly, two of these studies showed statistically significant improvements [19, 27], while Carey et al. [18] reported negative outcomes. This might be partly explained by the use of Box and Block test (B&B) as one outcome measure. As the B&B measures unilateral gross manual dexterity [29], it may not be the most appropriate nor sensitive measure to detect specific changes to the finger and wrist tracking intervention. Another interesting finding from this systematic review was where studies measured balance, telerehabilitation was found to be not as effective as usual care. There are two likely explanations for this. First, when addressing balance, it is important that there are adequate safeguards present with the patient and therefore traditional models of care (such as a face to face rehabilitation) might be more appropriate. Second, patient requiring balance retraining may require higher level of physical assistance, which could not be provided by telerehabilitation. As for other outcomes, telerehabilitation was found to have generally positive effects. While much of these positive effects were limited to within-group improvements, it does highlight the potential for telerehabilitation to have a positive impact by increasing patient options for treatment.

### NHMRC body of evidence framework

Table 4 summarises the analysis of results using the NHRMC FORM framework. The body of evidence suggests there is consistent evidence to indicate telerehabilitation may be as effective as usual care rehabilitation. However, due to heterogeneity in the evidence base, caution is required when interpreting these results.

**Table 4:** NHMRC Body of Evidence Framework.

Component	Grade	Comments
1. Evidence Base	B—*Good* *One or two level II studies with low risk of bias/several level III studies with low risk of bias*	Quantity: 11 studies Participants: 273 stroke survivors Level II: 11
2. Consistency	C—*Satisfactory* *Some inconsistency reflecting genuine uncertainty around clinical question*	All studies reported statistical significance Study Design: 7 RCTs, 3 Pilot RCTs Heterogeneous interventions Varied outcome measures taken at different time points
3. Clinical Impact	C—*Satisfactory* *Moderate*	Diverse interventions used, many difficult to replicate due to individually developed software/equipment No adverse effects reported 9 studies reported clinical importance
4. Generalisability	B—*Good* *Population(s) studied in body of evidence are similar to target population*	Population studied in evidence base is similar to target population Age range 28–85 years Studies conducted in 7 different countries
Grade of recommendations	C–*Satisfactory* *Body of Evidence provides some support for recommendations(s) but care should be taken in its application*.	Overall, most studies were of modest quality While there were consistent findings, the current evidence base lacks intervention criteria and outcome homogeneity for telerehabilitation and stroke.

## Discussion

The aim of this systematic review was to examine the effectiveness of telerehabilitation across a range of primary and secondary outcomes in the management of stroke patients. Given the increasing role of technology in health care and the promises it offers in ameliorating barriers to access, this review aimed to inform clinical practice and assist decision making for patients and health care professionals alike. A moderate body of evidence, consisting of 13 RCTs was identified. The summarised findings from this review suggest that telerehabilitation may be as effective as usual care for motor function, ADLs, independence and satisfaction/ quality of life. This positive finding may also extend to patient perspective measured in the form of patient satisfaction. Despite these positive findings, an unequivocal recommendation supporting telerehabilitation cannot be made due to methodological concerns and heterogeneity of interventions and outcomes. Furthermore, while these positive findings were mostly limited to within-group changes (and not consistently sustained for between-group changes), it does highlight the potential for telerehabilitation to be an alternate model of care when compared to usual care. Therefore, telerehabilitation may not be superior to, but as it is comparable with, traditional models of care, it may offer other benefits in practice settings (such as greater access to care for patients and improved efficiency for health professionals).

The results of this review are comparable to previous systematic reviews exploring the effectiveness of telerehabilitation for stroke [10] and Multiple Sclerosis (MS) [30]. While Laver et al. [10] did not provide specific recommendations for clinical practice from the Cochrane systematic review, due to methodological concerns and heterogeneity of interventions, they did flag important theoretical advantages for telerehabilitation to be used in addition to/instead of current stroke rehabilitation therapies. Our systematic review builds on the work by Laver et al. [10] as both reviews share seven common studies. However, given that this systematic review has identified six additional studies published since the conduct of Laver et al. [10], underpinned by a comprehensive search strategy involving black and grey literature, and used a different form of evidence synthesis (using the well-established and widely recognised NHMRC FORM framework), recommendations for clinical practice can be made. Findings of our systematic review are congruent with that of the Cochrane review by Khan et al. [30]. Findings from that systematic review indicate that despite low quality evidence base, telerehabilitation for people with MS did reduce short term disability and improve quality of life in the long term. Similar to this review, Khan et al. [30] also shared concerns regarding the methodological quality of the evidence base and its impact on making unequivocal recommendations.

The positive impact of telerehabilitation on motor function has been supported by other literature that also found positive outcomes [31, 32]. A systematic review by Langhorne, Coupar and Pollock [31] investigated the effect of common rehabilitation methods on motor recovery. They found positive outcomes for interventions such as constraint induced movement therapy, task specific training and training with a moving platform. Similarly, an RCT by Vahlberg et al. [32] investigated the effectiveness of progressive resistance and balance training for patients with chronic stroke, which resulted in improvement in motor function. An interesting finding in this systematic review was the lack of impact of telerehabilitation on balance. Previous research highlights the positive impact of face to face rehabilitation in improving balance [33, 34] which may mean telerehabilitation may not be the ideal model of care, if balance was the primary outcome of interest. Another explanation for this finding might be the intervention parameters used in the studies which measured balance. The three studies that reported findings favouring face to face rehabilitation for balance used varying telerehabilitation training regimes ranging from three sessions per week to daily [23, 24, 28]. This is contrary to the recommendation by the 2017 Stroke Foundation Guidelines [35] for minimum three hours per day of scheduled therapy, which may have resulted in findings favouring the usual care group.

Given the nature of the patient population, there was a great deal of diversity within the included studies on participant characteristics. For example, the time-period post-stroke when the telerehabilitation commenced varied greatly, with some studies including patients within few months of stroke [16, 17, 23, 28] while all other studies only commenced rehabilitation after at least five months. This variability might be explained due to varied needs of people with stroke, with some requiring intensive inpatient rehabilitation while others may access the same services from community settings [35]. Interestingly, the mean age of participants for six included studies was below 65 years of age [19, 20, 22, 23, 25, 26]. This contrasts with recent research which indicates that 70% of people experience their first stroke over 65 years of age [36]. It is unclear why there was an age-bias in the research literature on this topic. A possible explanation for excluding older people with stroke in telerehabilitation studies might be researchers’ perception that older people may experience discomfort or lack confidence with the use of technology and its impact on the effectiveness of telerehabilitation. Previous research supports the notion that the age of the patient does determine the stroke care they receive [37].

One of the challenges encountered in this systematic review was the use of telerehabilitation as an umbrella term for varied rehabilitation methods delivered via technology. While the use of technology was the common feature, there was a great deal of heterogeneity in terms of how the technology was utilised. This is supported by previous literature on this topic [10, 30, 31]. While some studies utilised specialised equipment for this purpose, other studies used commonly available tools (such as mobile phones and Skype). Similarly, the parameters (intensity, duration, frequency) underpinning telerehabilitation also varied significantly. While all telerehabilitation sessions were supervised, the person involved in the supervision process varied. There were no explicit justifications regarding the decision-making process on how the technology was utilised, which indicates these decisions were likely driven by contextual (availability of resources) and health professional (knowledge, skills and competence in technology use) reasons.

### Limitations

As with any research, there are some limitations to this systematic review. While this systematic review process was underpinned by best practice in the conduct of systematic reviews (PRISMA), publication and language bias are likely and hence should be acknowledged. Strategies to avoid publication bias included searching grey literature and secondary sources (which were used for pearling purposes). Despite this, it is possible that due to accessibility, and the imprecise nature of searching, in particular grey literature, some publications may have been missed. Language bias was present as this systematic review only included English-language publications. Given the search strategy was extensive and this resulted in seven publications from countries where English is not the first language means the impact of language bias has been minimised. As there is no universally-agreed definition of telerehabilitation, there was a great deal of heterogeneity in how telerehabilitation was delivered and its effectiveness measured. Many of the studies also failed to provide adequate descriptions of the interventions. Given that stroke affects the entire body, it is understandable that different studies explored different outcomes of interest. Furthermore, in some instances, such as patient satisfaction, the psychometric properties of the outcome measures were poorly reported. Due to these reasons, direct comparison of results between the studies was not possible. Other methodological concerns include maturation bias (lack of a true control group) and co-intervention bias (potentially unavoidable due to nature of stroke which requires multidisciplinary input). Finally, while this review did not set out to focus on chronic stroke patients, perhaps due to the nature of the intervention (telerehabilitation), many of the included studies did have a focus on this population. Given this to be the case, generalizability of these findings to other stroke patient groups (acute and sub-acute stroke patients) is limited.

## Conclusion

### Implications for clinical practice

Telerehabilitation is becoming increasingly popular as it is seen to promote equitable access to rehabilitation for populations with barriers to accessing traditional models of care. This review has identified a moderate body of consistent evidence indicating that telerehabilitation may be as effective as usual care for motor function, ADLs, independence, satisfaction/ quality of life and other miscellaneous outcomes. While these are encouraging findings, there are a range of factors to consider when implementing telerehabilitation in clinical practice. First, telerehabilitation requires dedicated resources and infrastructure in terms of equipment, network, training and setup. Second, health professionals may need specialised training, ongoing support and practical experience in delivering telerehabilitation and therefore this model of care may need to be delivered by experienced health professionals. Third, patient perspectives and preferences must also be considered as it is likely that some patients may feel uncertain about or perceive telerehabilitation as inferior model of care to traditional models of care (such as face to face rehabilitation). Finally, due to lack of clarity in the literature, no specific recommendation regarding the ideal timing and parameters underpinning telerehabilitation can be made.

### Implications for further research

A moderate body of consistent evidence has identified some positive benefits of telerehabilitation for people with stroke. Despite 13 RCTs contributing to this evidence base, some significant methodological concerns have highlighted the need for further research. Future research would benefit from developing standardised definition of and the parameters underpinning telerehabilitation. This will help to unpack the “black box” of telerehabilitation enabling concrete recommendations to be made regarding best practice. Similarly, methodologically sound RCTs using standardised intervention parameters and outcome measures will enable comparisons across homogenous studies. Finally, a number of important knowledge gaps at the practice level, (such as the training needs of the health professionals, required infrastructure, ongoing maintenance costs and patient preferences) should also be the focus of ongoing future research on this topic.

## Supporting information

S1 Table Intervention parameters. (DOCX)

S2 Table PRISMA checklist. (DOC)

S1 File Protocol. (PDF)

S1 Fig (TIF)
