# Supplementary material for: Effectiveness of telerehabilitation in the management of adults with stroke: A systematic review
Source: PLoS One. 2019 Nov 12;14(11):e0225150. doi: 10.1371/journal.pone.0225150 (PMC6850545; doi:10.1371/journal.pone.0225150)
Supplement: S1 Table — (DOCX) [file pone.0225150.s001.docx]

**S1 Table. Intervention parameters**

| **Study** | **Summary of intervention** | **Mode of tele communication** | **Supervision (Y/N)** | **Intensity** | **Frequency/Week** | **Duration** | |
| --- | --- | --- | --- | --- | --- | --- | --- |
|  |  |  |  |  |  | **Time (min)** | **Weeks** |
| Carey et al. 2007 | Flexion/Extension (index MCP joint & wrist).  Track group: feedback and accuracy score.  Move group: movement only. | Teleconferencing; with the use of a cellular phone and web camera. | Y* (5/10 sessions supervised). | 180 trials p/day for 10 days | Daily | Completed over 2-8 hours depending on rest breaks determined by participant | 10 days |
| Chen et al. 2017 | 1. Physical exercises (Passive UL stretches, motor imagery therapy, standing and balance training, gait training, assistance with ADLs).  2. ETNMS | Live videoconferencing system (muscle electricity biofeedback instrument and physiological data collection system at patient end) | Y* (ETNMS until patient/caregiver competent). | NAD | Daily | Physical Exercises: 1 hour ETNS: 20 minutes | 12 weeks |
| Chumbler et al. 2012 | 1. Home Visits 2. Telephone interventions 3. In home messaging device | Videotaping and analog phone lines were used. | Y* (3 home visits supervised). | NAD | Week 1: Home visit 2: Phone Call 3: Home visit  4: Phone call 5: Home visit  6: Phone call 8: Phone call 12: Phone call IMHD: Daily  Exercise frequency: NAD | Home visits: 1 hour Phone calls: NAD Exercise duration: NAD | Total: 12 weeks |
| Chumbler et al. 2015 | Please see Chumbler et al. 2012 for all details of intervention |  |  |  |  |  |  |
| Deng et al. 2012 | Dorsiflexion/plantarflexion (talocrural joint)  Track group: feedback and accuracy score.  Move group: movement only. | Teleconferencing. A remote desktop application (LogMeIn) and Skype with webcams (Logitech C905) | Y* (2 sessions per week, daily performance emails). | 180 repetitions per day/ 20 days | Daily | Approx. 60 minutes | 3 weeks (20 days) |
| Forducey et al. 2012 | Intervention sessions aimed at retraining of self-care, mobility and posture, and home modiﬁcations. | delivered via desktop videophone using standard telephone lines. | Y | NAD | 2 days per week | NAD | 6 weeks |
| Huigjen et al. 2008 | Training via the telerehabilitation system: Home Care Activity Desk (HCAD) for 4 weeks aimed at functional upper limb training | Hospital-based server and a portable unit installed at home | Y* (weekly videoconference review, daily videos uploaded to server) | NAD | 5 days per week | 30 minutes | 4 weeks |
| Kizony et al. 2013 | Game based therapy (puzzle, memory, pizza/hamburger, arrows and tasks).  Reaching movements of ULs, avoiding compensatory shoulder, elbow and trunk motions. | Gertner Tele Motion Rehab (TMR) system set up (Weiss 2012 describes in detail) with a 3D video capture camera-based system - Kinect camera and software | Y (therapist present 3/12 sessions, 8/12 via telerehabilitation system) | Ranging from simple to difficult, depending on game | 3 days per week | 45 minutes | 4 weeks |
| Krpic, Savanovic and Cikajlo 2013 | Virtual balance training using a standing frame to facilitate an upright posture. | Standing frame (BT), specific programs and computer to simulate the virtual environment | Y* (Therapist assistance for weeks 1 and 2. Final week unsupervised or non-professional assistance) | NAD | 5 days per week | 15 minutes per day | 3 weeks |
| Lin et al. 2014 | 1. Static and Dynamic sitting balance.  2. Sitting balance on foam surface.  3 and 4. Static and dynamic standing balance.  (At each stage, participants had to manipulate objects on a touch screen with ULs) | Logitech web camera, wireless sensor network | Y (therapist supervision via telerehabilitation system, volunteer or non-medical person present with patient for safety and assistance) | light to moderate exercise intensity (Borg scale 12–14) | 3 sessions per week | 50 minutes | 4 weeks |
| Llorens et al. 2015 | VR balance intervention (participants required to reach for objects in virtual environment with one foot, while maintaining positon of other foot). | A television, a conventional computer, and a Microsoft Kinect | Y* (telerehabilition intervention was not supervised, co-intervention was supervised) | NAD | 3 sessions per week. Six, 6-minute repetitions with 90-second breaks | 45 minutes | 6.5 weeks |
| Piron et al. 2008 | VR tasks (for compromised arm). Participants could see their movement along with ideal prerecorded movement from therapist | 3D motion tracking system (Polhemus 3Space Fastrak, Vermont, US) | Y (therapist interaction via telerehabilitation system) | NAD | Daily | 1 hour per day | 4 weeks |
| Piron et al. 2009 | 5 simple, VR tasks (for compromised arm).  Participants moved a real object following a virtual trajectory on the screen | 3D motion tracking system (Polhemus 3Space Fastrak, Vermont, US) | Y (specific task feedback via telerehabilition system from therapist) | NAD | 5 days per week | 1 hour per day | 4 weeks |

**Legend =** MCP = Metacarpal phalangeal joint, Y = Yes, N = No, * = Partial supervision, UL = Upper Limb, ADL = Activities of Daily Living, ETNMS = Electromyography-triggered neuro-muscular stimulation, VR = Virtual reality
