# Supplementary material for: Effectiveness of telerehabilitation in the management of adults with stroke: A systematic review
Source: PLoS One. 2019 Nov 12;14(11):e0225150. doi: 10.1371/journal.pone.0225150 (PMC6850545; doi:10.1371/journal.pone.0225150)
Supplement: S1 File — (PDF) [file pone.0225150.s003.pdf]

Evidence of effects of telerehabilitation on function compared to usual model of care in the management of stroke patients: a systematic review

Emma Appleby, Sophie Gill, Lucinda Hayes, Tessa Walker, Matt Walsh, Saravana Kumar

Citation

Emma Appleby, Sophie Gill, Lucinda Hayes, Tessa Walker, Matt Walsh, Saravana Kumar. Evidence of effects of telerehabilitation on function compared to usual model of care in the management of stroke patients: a systematic review. PROSPERO 2018 CRD42018090445 Available from: http://www.crd.york.ac.uk/PROSPERO/display_record.php?ID=CRD42018090445

Review question

What effect does telerehabilitation have on function, independence, activities of daily living and and other outcomes, when compared to conventional model of care, in the management of stroke patients?

Searches

The following electronic databases will be searched: MEDLINE Ovid; Embase; Emcare; Scopus; The Cochrane Library; PEDro, OTSeeker. The references of relevant articles will be searched to identify potential additional articles (pearling). Grey literature searching through an internet web engine (Google and Google Scholar) will be undertaken to identify any additional publications. Websites of relevant organisations (such as the National Stroke Foundation) will also be searched for additional publications. All databases will be searched from inception to current. Language (English) and human only restrictions will also be applied.

Search strategy

https://www.crd.york.ac.uk/PROSPEROFILES/90445_STRATEGY_20180306.pdf

Types of study to be included

This review will include all primary research of quantitative research paradigm including randomised controlled trials, clinical trials, case-controls, cohort studies, pilot studies and case reports/case series. This review will exclude secondary research, protocol studies, opinion papers, editorials, conference presentations and research from the qualitative research paradigm.

Condition or domain being studied

The purpose of this systematic review is to evaluate the effectiveness of telerehabilitation in regard to a change in patient function, independence and activities of daily living. This review will specifically focus on stroke patients using telerehabilitation services.

Participants/population

Inclusion: Adult stroke patients; human.

Exclusion: Children.

Intervention(s), exposure(s)

Inclusion: Rehabilitation with health professional/s delivered and/or conducted via videoconferencing technology;

Also, commonly referred to as Telerehabilitaton, E-rehabilitation, Mhealth or telemedicine.

Exclusion: Virtual reality used for rehabilitation without therapist input, video games used for rehabilitation without therapist input, therapy delivered only via telephone consultations without video conferencing.

Comparator(s)/control

Inclusion: Usual care; face to face rehabilitation, conventional rehabilitation.

Exclusion: Medical Interventions.

Context

Main outcome(s)
Function and activities of daily living

Additional outcome(s)
Including but not limited to: independence and patient satisfaction

Data extraction (selection and coding)
The titles generated by the electronic databases will be scanned by two researchers to identify potentially relevant papers. Where the titles would not allow determination of relevance to the topic, abstracts will be reviewed. If the title and abstracts meet the inclusion criteria for this review, they will be initially selected to be part of the review (step one). In step two, full-text copies of eligible articles will be retrieved for full examination. During this process, the complete papers will be examined to identify if it meets the inclusion criteria for this review. Publications, which meet all the inclusion parameters, will be included in this review. Publications, which do not meet the inclusion parameters, will be excluded from the review process. Any disagreements in determining the inclusion/exclusion of paper will be resolved through discussion. Customized data extraction forms will be developed specifically for this systematic literature review and will contain key elements, which are pertinent to address the objectives and questions of this review.

Risk of bias (quality) assessment
Once relevant research publications have been identified, we will evaluate the methodological quality of selected publications. We will use the McMaster Critical Appraisal Tools (Quantitative Studies) to assess the quality of the included studies. This will be undertaken by two independent reviewers and any differences will be addressed through discussion with a third independent reviewer. Studies will not be excluded based on the quality score. However, this information will be used to report, analyse and discuss the overall review findings

Strategy for data synthesis
Pooling of data by meta-analysis will be undertaken where similar populations, interventions, outcomes and designs are found and the research team determines if the heterogeneity is low. Heterogeneity will be assessed through methodological means as well as subjectively and formally through I^2 statistic. I^2 statistic values of 25%, 50% and 75% represent low, moderate and severe heterogeneity. For the purpose of this review, if the I^2 statistic value was greater than 50%, then readers will be notified of substantial heterogeneity and cautioned regarding interpreting aggregated results.

To determine the impact of the intervention compared to the control condition, the mean difference or standardised mean difference (SMD) and 95% confidence interval (95% CI) will be calculated for each reported outcome using Cochrane Review Manager (V.5). To calculate SMDs the difference in mean scores between the intervention and control condition will be divided by the pooled standard deviation. SMDs will be considered statistically significant if their 95% CI does not cross zero. Interpretation of the strength of the SMD statistics will be based on guidelines suggested by Cohen where a small effect equal or less than 0.2, medium effect is equal or greater than 0.5 and a large effect is equal or greater than 0.8).

If a meta-analysis is not possible, descriptive synthesis of the included studies will be conducted. The NHMRC FORM framework (Hillier et al 2011) will be used to underpin this process. The NHMRC FORM methodology considers all evidence dimensions of all studies, which are used in the development of a specific recommendation. There are five key components, 1) quantity and quality of evidence; 2) consistency; 3) clinical impact; 4) generalisability; 5) applicability to the Australian health-care setting. The applicability component was not used in this review, given the findings may be relevant to an international population. This framework allowed evidence-based recommendations for future research and clinical practice to be made.

Analysis of subgroups or subsets

None Planned

Contact details for further information

Saravana Kumar
Saravana.kumar@unisa.edu.au

Organisational affiliation of the review

University of South Australia
www.unisa.edu.au

Review team members and their organisational affiliations

Ms Emma Appleby. University of South Australia
Ms Sophie Gill. University of South Australia
Ms Lucinda Hayes. University of South Australia
Ms Tessa Walker. University of South Australia
Mr Matt Walsh. University of South Australia
Dr Saravana Kumar. University of South Australia

Type and method of review

Systematic review

Anticipated or actual start date

25 March 2018

Anticipated completion date

31 December 2018

Funding sources/sponsors

No Funding

Conflicts of interest

Language

(there is not an English language summary)

Country

Australia

Stage of review

Review Ongoing

Subject index terms status

Subject indexing assigned by CRD

Subject index terms

Humans; Stroke; Stroke Rehabilitation; Telerehabilitation

Date of registration in PROSPERO

09 March 2018

Date of publication of this version

09 March 2018

Details of any existing review of the same topic by the same authors

Stage of review at time of this submission

Stage	Started	Completed
Preliminary searches	Yes	No
Piloting of the study selection process	Yes	No
Formal screening of search results against eligibility criteria	No	No
Data extraction	No	No
Risk of bias (quality) assessment	No	No
Data analysis	No	No

Versions

09 March 2018

PROSPERO
This information has been provided by the named contact for this review. CRD has accepted this information in good faith and registered the review in PROSPERO. The registrant confirms that the information supplied for this submission is accurate and complete. CRD bears no responsibility or liability for the content of this registration record, any associated files or external websites.
