# Supplementary figures and images for: Effectiveness of telerehabilitation in the management of adults with stroke: A systematic review
Source: PLoS One. 2019 Nov 12;14(11):e0225150. doi: 10.1371/journal.pone.0225150 (PMC6850545; doi:10.1371/journal.pone.0225150)

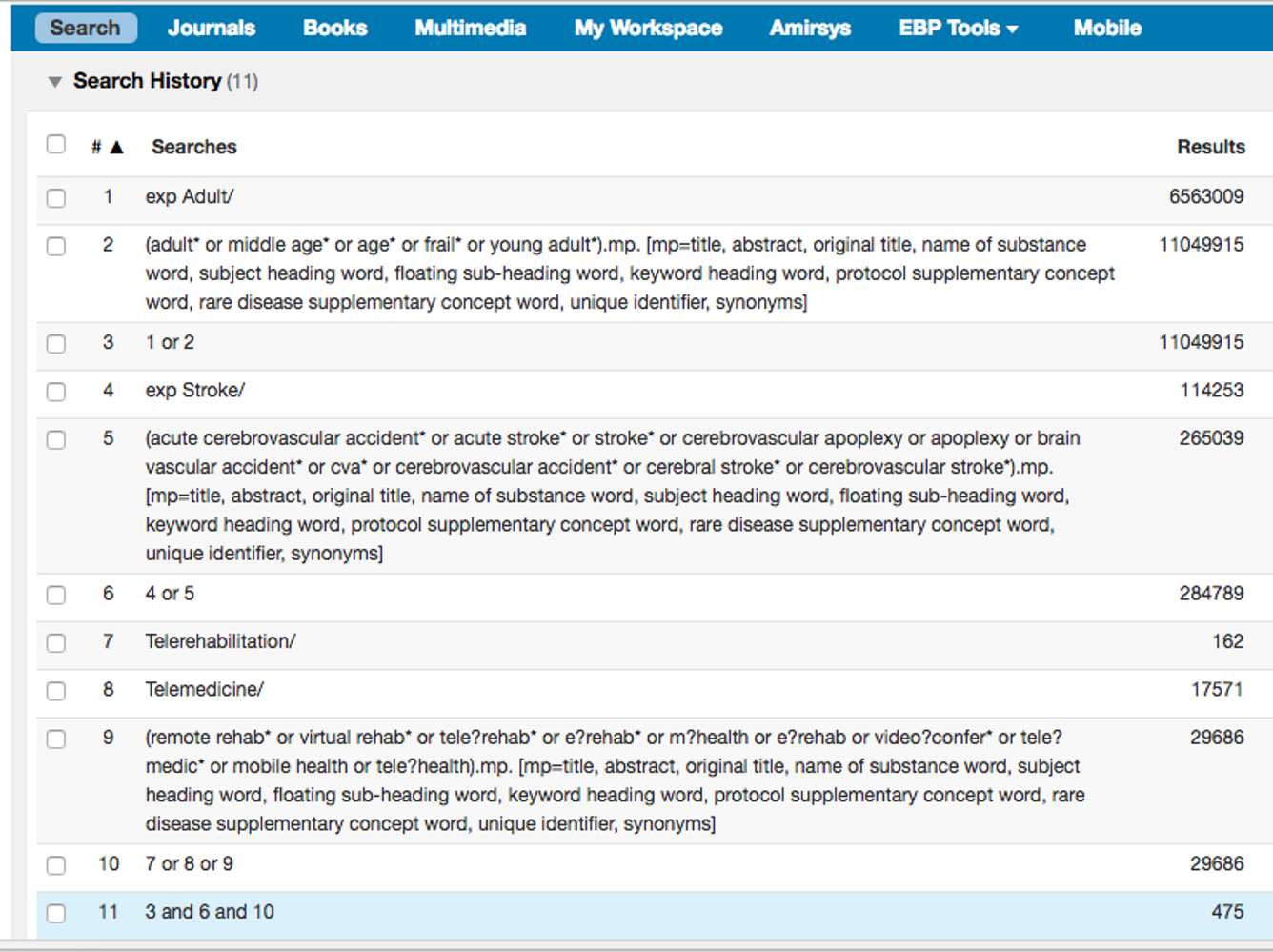

Supplement: S1 Fig — (TIF) [file pone.0225150.s004.tif]
